# ZBP1 contributes to IFN-β-induced PANoptosis-related skeletal muscle cell injury in dermatomyositis

**DOI:** 10.3389/fimmu.2026.1955536

**Published:** 2026-09-24

**Authors:** Ziheng Zhu, Ruofan Liu, Yuxiang Xu, Ruxuan Sheng, Chuanbing Huang

**Affiliations:** 1 Department of Rheumatology and Immunology, The First Affiliated Hospital of Anhui University of Chinese Medicine, Hefei, China; 2 Key Laboratory of Xin’an Medicine, Ministry of Education, Anhui University of Chinese Medicine, Hefei, China

**Keywords:** baicalin, dermatomyositis, machine learning, PANoptosis, programmed cell death, ZBP1

## Abstract

**Background:**

Dermatomyositis (DM) is a systemic autoimmune disease characterized by cutaneous manifestations and inflammatory skeletal muscle injury. Dysregulated type I interferon signaling and regulated cell death pathways may converge to promote myofiber damage. PANoptosis integrates core components of pyroptosis, apoptosis, and necroptosis, but its contribution to DM remains unclear.

**Methods:**

Transcriptomic datasets from DM skin and skeletal muscle were integrated to identify differentially expressed genes overlapping with a predefined PANoptosis-related gene set. Candidate core genes were prioritized using least absolute shrinkage and selection operator regression, random forest, and extreme gradient boosting. A four-gene PANoptosis-related risk score (PRS) was constructed and evaluated for discriminatory performance and associations with inferred immune cell composition. Adult human skeletal muscle cells (HSkMCs) were exposed to interferon-β (IFN-β), and PANoptosis-associated injury was assessed by transmission electron microscopy, western blotting, co-immunoprecipitation, Cell Counting Kit-8 assays, LDH release, and ROS measurement. Molecular docking and microscale thermophoresis (MST) were used to evaluate baicalin binding to ZBP1.

**Results:**

Eleven DM-associated PANoptosis-related genes were identified, and ZBP1, CD14, TLR3, and TNFSF10 were consistently prioritized. The PRS yielded an area under the curve of 0.9375, and high-PRS samples showed stronger innate immune and inflammatory transcriptional signatures. All four genes correlated positively with the inferred abundance of M1 macrophages and were upregulated in peripheral blood mononuclear cells from patients with DM. ZBP1 expression correlated positively with serum creatine kinase. IFN-β reduced HSkMC viability, increased LDH release, and induced ultrastructural and molecular changes associated with apoptosis, pyroptosis, and necroptosis. ZBP1 overexpression increased its association with RIPK3, caspase-8, and caspase-6, whereas ZBP1 knockdown attenuated PANoptosis-associated signaling, ROS accumulation, LDH release, and viability loss. MST confirmed direct baicalin–ZBP1 binding. Baicalin reduced ZBP1-associated protein co-precipitation and alleviated IFN-β-induced cellular injury.

**Conclusion:**

This study identified four candidate core PANoptosis-related genes associated with DM and developed a four-gene PRS model with strong discriminatory performance for distinguishing DM samples from controls. Mechanistically, ZBP1 may contribute to IFN-β-induced PANoptosis-like injury in HSkMCs by facilitating its association with RIPK3, caspase-8, and caspase-6. Baicalin may protect HSkMCs by directly binding to ZBP1 and attenuating these PANoptosis-associated protein interactions.

## 1 Introduction

Dermatomyositis (DM) is a systemic autoimmune disease characterized by distinctive cutaneous manifestations and inflammatory involvement. Patients commonly present with proximal muscle weakness and elevated serum muscle enzyme levels and may also develop extramuscular complications, particularly interstitial lung disease (1, 2). The clinical manifestations of DM are highly heterogeneous. Approximately 20% of patients have clinically amyopathic or hypomyopathic DM, with absent or minimal muscle weakness and potentially normal serum creatine kinase (CK) levels (3, 4). Current classification criteria and conventional measurements of serum muscle enzymes may therefore have limited sensitivity for early disease recognition and may not adequately reflect longitudinal changes in disease activity (5, 6). Although muscle biopsy provides valuable pathological information, its invasive nature and susceptibility to sampling error limit its suitability for repeated assessment (7). These limitations underscore the need for non-invasive biomarkers that facilitate early diagnosis and longitudinal monitoring while reflecting the underlying pathogenic processes in DM. Biomarkers linked to immune and inflammatory responses and programmed cell death are of particular interest because these processes are increasingly implicated in DM pathogenesis.

The pathogenesis of DM is multifactorial, involving genetic susceptibility, dysregulated innate and adaptive immune responses, complement activation, microvascular injury, and sustained inflammation (8–10). The conventional vascular model proposes that complement-mediated endothelial injury and capillary loss impair tissue perfusion, leading to ischemic damage and perifascicular myofiber atrophy (11). However, many clinical and basic studies have found that DM muscle tissues also exhibit robust activation of type I interferon signaling, inflammatory cell infiltration, and variable degrees of myofiber degeneration and necrosis (12–14). These findings suggest that microvascular ischemia alone does not fully account for the complex tissue injury observed in DM. Emerging evidence further indicates that skeletal muscle cells are not passive targets of immune-mediated damage. Skeletal muscle cells may actively respond to inflammatory signals and engage programmed cell death (PCD) pathways (15–17). Apoptosis, pyroptosis, and necroptosis have each been implicated in DM-associated muscle injury (18, 19), but whether these pathways are coordinately activated remains unclear.

In this context, PANoptosis offers an integrative conceptual framework for understanding how multiple cell death programs may converge during DM-associated tissue injury. It is an inflammatory form of PCD involving coordinated engagement of pyroptotic, apoptotic, and necroptotic machinery through PANoptosome-associated signaling (20). Unlike any single cell death pathway considered in isolation, PANoptosis may more comprehensively account for the concurrent activation of distinct cell death signals, persistent amplification of inflammation, and progressive tissue injury in autoimmune diseases (21).

Nevertheless, whether PANoptosis contributes to skeletal muscle injury in DM, which molecular regulators are central to this process, and whether these regulators represent therapeutically actionable targets remain unclear.

Baicalin is a major bioactive flavonoid derived from the traditional Chinese medicinal plant *Scutellaria baicalensis* Georgi and has anti-inflammatory, antioxidant, and immunomodulatory properties (22, 23). Previous studies suggest that baicalin can modulate apoptosis, pyroptosis, and necroptosis and attenuate inflammatory responses and tissue injury through signaling pathways linked to PANoptosis (24–26). However, it remains unknown whether baicalin directly targets key regulators of PANoptosis, modulates PANoptosome-associated protein interactions, and protects skeletal muscle cells against PANoptosis-like injury in the context of DM.

In this study, we integrated skin and skeletal muscle transcriptomic datasets from patients with DM and applied differential expression, functional enrichment, and machine-learning analyses to identify PANoptosis-related candidate genes and construct a PANoptosis-related risk score (PRS). Immune cell infiltration analysis and expression profiling of the candidate genes in peripheral blood mononuclear cells (PBMCs) from patients with DM were subsequently performed to assess their immunological and clinical relevance. To investigate the functional role of the key target, adult human skeletal muscle cells (HSkMCs) were exposed to interferon beta (IFN-β), followed by gene knockdown, overexpression, and co-immunoprecipitation (Co-IP) analyses of PANoptosis-associated signaling. Finally, molecular docking and microscale thermophoresis (MST) were used to characterize the binding of baicalin to the key target, whereas cell-based functional assays were performed to evaluate its protective effects against IFN-β-induced HSkMC injury.

## 2 Methods

### 2.1 Dataset acquisition and identification of DM-associated PANoptosis-related genes

The DM-related transcriptomic datasets GSE46239 and GSE143323 were obtained from the Gene Expression Omnibus database. GSE46239 comprises skin tissue samples from patients with DM and control individuals, whereas GSE143323 comprises skeletal muscle tissue samples from patients with DM and control individuals. A curated set of 109 PANoptosis-related genes was compiled from previously published studies (27), and the complete gene list is provided in **Supplementary Table 1**.

Each dataset was analyzed separately for differential gene expression using GEO2R, which applies the limma statistical framework. Genes with P < 0.05 and an absolute log2 fold change (|log2FC|) > 0.58 were considered differentially expressed genes (DEGs). Genome-wide differential expression patterns were visualized using volcano plots. DEGs shared between the two datasets and exhibiting concordant directions of change were then identified by separately intersecting the upregulated and downregulated gene sets. These common DEGs were subsequently intersected with the curated set of 109 PANoptosis-related genes to identify candidate PANoptosis-related genes associated with DM. Functional enrichment analyses of the candidate genes were performed using the DAVID. Gene Ontology (GO) enrichment analysis was conducted across the biological process, molecular function, and cellular component categories. Pathway enrichment analysis was additionally performed to characterize the biological processes and signaling pathways potentially associated with the candidate PANoptosis-related genes.

### 2.2 Machine learning-based identification of candidate core genes

To identify candidate genes capable of distinguishing DM from control samples, three machine-learning algorithms were applied in parallel to the DM-associated candidate PANoptosis-related gene set: least absolute shrinkage and selection operator (LASSO) regression, random forest (RF), and extreme gradient boosting (XGBoost). LASSO regression was implemented using the glmnet package in R, and genes with nonzero coefficients at the optimal penalty parameter were retained (28). RF analysis was performed using the randomForest package, and candidate genes were ranked according to their variable-importance scores (29). XGBoost analysis was conducted using the xgboost package, with candidate genes ranked according to their feature importance scores (30). Five-fold cross-validation was used to optimize model-specific hyperparameters and evaluate model stability. Genes consistently selected by all three algorithms were defined as candidate core PANoptosis-related genes and retained for subsequent analyses.

### 2.3 Development of the PANoptosis-related risk score model

A PANoptosis-related risk score (PRS) model was developed using binary logistic regression (31). Disease status, classified as DM or control, was specified as the binary outcome, and the expression levels of the four candidate core genes were included as predictor variables. For each sample, the PRS was calculated using the following linear predictor:

PRS=−35.772+(3.971×Exp[ZBP1])+(2.574×Exp[CD14])−(3.927×Exp[TLR3])+(2.237×Exp[TNFSF10]).

### 2.4 Immune cell deconvolution and correlation analysis

The relative abundance of immune cell subsets in skeletal muscle samples from the GSE143323 dataset was estimated using the CIBERSORTx online platform and the leukocyte signature matrix 22 (LM22). LM22 distinguishes 22 human immune cell phenotypes, including subsets of B cells, T cells, natural killer cells, monocytes, macrophages, dendritic cells, mast cells, eosinophils, and neutrophils. The estimated proportions of these immune cell subsets were compared between the DM and control groups to characterize alterations in the immune-cell composition of DM skeletal muscle. Correlation analyses were subsequently performed between the expression levels of the candidate core PANoptosis-related genes and the estimated abundance of each immune cell subset to examine their potential relationships with the immune microenvironment in DM.

### 2.5 Study participants and clinical sample collection

The study protocol was approved by the Ethics Committee of the First Affiliated Hospital of Anhui University of Chinese Medicine (approval No. 2025AH-193-01). Written informed consent was obtained from all participants before sample collection. Between January and June 2026, 25 patients with DM were consecutively recruited from the Department of Rheumatology and Immunology at the First Affiliated Hospital of Anhui University of Chinese Medicine. During the same period, 15 age- and sex-matched healthy volunteers were recruited as healthy controls (HCs). Approximately 10 mL of peripheral venous blood was collected from each participant into ethylenediaminetetraacetic acid (EDTA)-containing tubes. Baseline characteristics of the DM and HC groups are shown in **Supplementary Table 2**.

### 2.6 Cell culture and establishment of the IFN-β-induced HSkMC injury model

Immortalized adult human skeletal muscle cells (HSkMCs; catalog No. HUM-iCell-0086a) were obtained from iCell Bioscience Inc. (Shanghai, China). Cryopreserved cells were rapidly thawed in a 37 °C water bath, transferred to prewarmed complete culture medium, collected by centrifugation, and seeded into culture flasks. Cells were maintained under standard culture conditions and passaged upon reaching the designated confluence. For routine passaging, cells were washed with phosphate-buffered saline (PBS), detached with trypsin, and neutralized with complete culture medium. The cell suspension was then collected by centrifugation, resuspended in fresh complete medium, counted, and reseeded at a split ratio of 1:2.

Based on the protocol reported by Zhang et al. (32), an *in vitro* HSkMC injury model was established by treating the cells with 10^4^ U/mL recombinant human interferon beta (IFN-β; catalog No. HY-P73128, MedChemExpress, New Jersey, USA) for 24 h. Control cells were cultured in the corresponding complete medium without IFN-β. To evaluate the potential cytotoxicity of baicalin under basal culture conditions, HSkMCs were exposed to 0, 10, 20, 40, 60, 80, or 100 μM baicalin for 6, 12, 24, or 48 h. Cell viability was subsequently assessed using the Cell Counting Kit-8 (CCK-8) assay. A suitable concentration and treatment duration for subsequent experiments were then selected by evaluating the effects of baicalin on cell viability in the IFN-β-induced HSkMC injury model.

### 2.7 Overexpression and knockdown of Z-DNA-binding protein 1

HSkMCs were seeded in six-well plates and transfected at approximately 70% confluence using Lipo8000™ transfection reagent (catalog No. C0533; Beyotime Biotechnology, Shanghai, China) according to the manufacturer’s instructions. For overexpression, cells were transfected with 4 μg per well of a pcDNA3.1(+)-ZBP1 overexpression plasmid or the corresponding empty pcDNA3.1(+) vector. For gene knockdown, cells were transfected with 100 pmol per well of one of three ZBP1-targeting small interfering RNAs (si-ZBP1) or negative-control small interfering RNA (si-NC). After a 4-h transfection period, the transfection medium was replaced with fresh complete culture medium. Cells were harvested at 48 h post-transfection, and ZBP1 mRNA and ZBP1 protein levels were measured by RT-qPCR and western blotting, respectively. The siRNA that produced the greatest reduction in ZBP1 expression was selected for subsequent experiments. The siRNA sequences are provided in **Supplementary Table 3**.

### 2.8 Western blot

Cells were harvested, and total protein was extracted using radioimmunoprecipitation assay (RIPA) lysis buffer supplemented with 1 mM phenylmethylsulfonyl fluoride (PMSF). Following protein quantification, equal amounts of protein (30 μg per lane) were separated on 10% sodium dodecyl sulfate-polyacrylamide gels and transferred onto polyvinylidene difluoride (PVDF) membranes. Membranes were blocked with 5% non-fat dry milk, whereas 5% bovine serum albumin (BSA) was used when phosphorylated proteins were detected. The membranes were subsequently incubated overnight at 4 °C with primary antibodies against ZBP1 (catalog No. 52538, Sciben; 1:1,000), caspase-6 (catalog No. 71164, Sciben; 1:1,000), caspase-8 (catalog No. AF6442, Affinity; 1:1,000), full-length gasdermin D and its N-terminal fragment (GSDMD-FL and GSDMD-N; catalog No. AF4012, Affinity; 1:1,000), receptor-interacting serine/threonine-protein kinase 3 (RIPK3; catalog No. 6145, Sciben; 1:1,000), phosphorylated RIPK3 (p-RIPK3; catalog No. U0959, Sciben; 1:1,000), and β-actin (catalog No. TA-09, Zs-BIO; 1:1,000).

After washing, the membranes were incubated for 2 h at room temperature with horseradish peroxidase-conjugated goat anti-rabbit immunoglobulin G (catalog No. ZB-2301, Zs-BIO; 1:10,000) or goat anti-mouse immunoglobulin G (catalog No. ZB-2305, Zs-BIO; 1:10,000), as appropriate for the host species of the primary antibody. Protein bands were visualized using enhanced chemiluminescence reagent (catalog No. GK10008, GLPBIO), and densitometric analysis was performed using ImageJ software. The levels of ZBP1, caspase-6, caspase-8, GSDMD-FL, GSDMD-N, and RIPK3 were normalized to β-actin.

### 2.9 Reverse transcription quantitative polymerase chain reaction

Total RNA was extracted from cells in each experimental group using TRIzol reagent (catalog No. SB-MR009; Share-bio Biotechnology Co., Ltd., Shanghai, China). One microgram of total RNA was reverse transcribed into complementary DNA (cDNA) using the All-in-One First-Strand Synthesis MasterMix with dsDNase (catalog No. EG15133S; iScience, Pennsylvania, USA) in a total reaction volume of 20 μL. Quantitative polymerase chain reaction (qPCR) was performed using Taq SYBR Green qPCR Premix (Universal) (catalog No. EG20117M; iScience, Pennsylvania, USA) on a StepOnePlus Real-Time PCR System (Applied Biosystems, California, USA). Each 20-μL reaction contained 10 μL of qPCR premix, 0.4 μL each of the forward and reverse primers (10 μM), 3 μL of cDNA template, and 6.2 μL of RNase-free water. The amplification program consisted of an initial denaturation at 95 °C for 30 s, followed by 40 cycles of denaturation at 95 °C for 15 s and annealing/extension at 60 °C for 30 s. Relative mRNA expression was calculated using the 2^−ΔΔCt^ method, with *ACTB* (β-actin) serving as the endogenous reference gene. All primers were synthesized by Sangon Biotech (Shanghai, China), and their sequences are provided in **Supplementary Table 4**.

### 2.10 Co-immunoprecipitation assay

Co-immunoprecipitation (Co-IP) was performed using a Protein A+G magnetic bead immunoprecipitation kit (catalog No. P2179S; Beyotime Biotechnology, Shanghai, China) to assess the association of hemagglutinin (HA)-tagged ZBP1 with PANoptosis-related proteins. Cells from each experimental group were lysed in immunoprecipitation lysis buffer supplemented with protease inhibitors. Cell lysates were clarified by centrifugation at 14,000 × g for 3–5 min at 4 °C, and the resulting supernatants were collected. An aliquot of each lysate was retained as the input fraction, and a species-matched immunoglobulin G (IgG) control was processed in parallel as a negative control. For immunoprecipitation, 4 μg of anti-HA antibody (catalog No. 51064-2-AP; Proteintech, Wuhan, China) was incubated with Protein A+G magnetic beads for 1 h at room temperature. After washing, the antibody-conjugated beads were incubated with the clarified cell lysates overnight at 4 °C with gentle rotation. The beads were subsequently washed and resuspended in 1× sodium dodecyl sulfate-polyacrylamide gel electrophoresis sample buffer, followed by heating at 95 °C for 5 min to elute the immunoprecipitated proteins. Western blotting was performed using primary antibodies against HA (catalog No. 51064-2-AP, Proteintech; 1:5,000), caspase-6 (catalog No. 71164, Sciben; 1:1,000), caspase-8 (catalog No. AF6442, Affinity; 1:1,000), and RIPK3 (catalog No. 6145, Sciben; 1:1,000). After incubation with the appropriate horseradish peroxidase-conjugated secondary antibodies, protein bands were detected using enhanced chemiluminescence reagent (catalog No. 34094; Thermo Fisher Scientific). Densitometric analysis was performed using ImageJ software.

### 2.11 Cell counting kit-8 assay

Cells from each experimental group were harvested, resuspended in complete culture medium, and gently mixed to obtain a uniform single-cell suspension. The cells were seeded into culture plates in 100 μL of complete medium per well. Peripheral wells not used for cell culture were filled with an equal volume of sterile phosphate-buffered saline (PBS) to minimize evaporation-associated edge effects. After overnight incubation at 37 °C in a humidified atmosphere containing 5% carbon dioxide (CO_2_), 10 μL of CCK-8 reagent (catalog No. GK100001; GLPBIO, California, USA) was added to each well. The plates were incubated for an additional 1 h, after which absorbance was measured at 450 nm using a microplate reader. Wells containing complete culture medium and CCK-8 reagent but no cells were used as blanks.

### 2.12 Fluorescence-based detection of intracellular reactive oxygen species

Following the indicated treatments, cells were washed with phosphate-buffered saline (PBS) and incubated with 2′,7′-dichlorodihydrofluorescein diacetate (DCFH-DA) working solution, prepared by diluting the probe 1:1,000 in serum-free medium, using a reactive oxygen species (ROS) assay kit (catalog No. S0033S; Beyotime Biotechnology, Shanghai, China). Cells were incubated with the probe for 30 min at 37 °C in the dark, with gentle agitation at intervals during incubation. The cells were then washed three times with serum-free medium to remove residual extracellular probe. Fluorescence images were acquired using an inverted fluorescence microscope under identical imaging settings for all experimental groups. Three randomly selected, non-overlapping fields were imaged for each sample. Intracellular ROS accumulation was assessed on the basis of the relative green fluorescence intensity.

### 2.13 Microscale thermophoresis

Recombinant ZBP1 protein was fluorescently labeled using the RED-NHS Protein Labeling Kit (catalog No. MO-L011; NanoTemper Technologies) according to the manufacturer’s instructions, followed by removal of excess free dye. Baicalin was prepared as a 16-point twofold serial dilution and mixed at a 1:1 volume ratio with the fluorescently labeled ZBP1 protein, with the final protein concentration kept constant across all samples. The mixtures were incubated for 20 min at room temperature in the dark and then loaded into standard capillaries (catalog No. MO-K022; NanoTemper Technologies). Microscale thermophoresis measurements were performed using a Monolith X instrument (NanoTemper Technologies, Germany). Binding curves were generated and fitted using NanoTemper analysis software, and the equilibrium dissociation constant (K_D_) was determined from the fitted concentration-response data.

### 2.14 Statistical analysis

Statistical analyses were performed using GraphPad Prism version 10.1.2 (GraphPad Software, California, USA). Quantitative data are presented as the mean ± standard deviation (SD). For normally distributed data with equal variances, comparisons between two independent groups were performed using an unpaired Student’s t-test, whereas comparisons among three or more groups were performed using one-way analysis of variance (ANOVA), followed by Tukey’s multiple-comparisons test. When the assumption of equal variances was not met, Welch’s t-test was used for comparisons between two groups, and Brown–Forsythe and Welch ANOVA followed by Dunnett’s T3 multiple-comparisons test was used for comparisons among three or more groups. Associations between continuous variables were evaluated using Spearman’s rank correlation coefficient. All statistical tests were two-sided, and a P value < 0.05 was considered statistically significant.

## 3 Results

### 3.1 Bioinformatics-based identification of candidate PANoptosis-related genes in dermatomyositis

We analyzed two transcriptomic datasets obtained from the Gene Expression Omnibus database to identify PANoptosis-related genes potentially involved in dermatomyositis (DM). GSE46239 comprised skin samples from 48 patients with DM and 4 control individuals, whereas GSE143323 comprised skeletal muscle samples from 39 patients with DM and 20 control individuals. Differential expression analysis was performed independently in each dataset, followed by identification of genes exhibiting concordant changes across the two tissues. A total of 304 genes were consistently upregulated and 10 were consistently downregulated in both DM skin and skeletal muscle samples (**Figures 1A, B**). These findings revealed a shared cross-tissue transcriptional signature associated with DM.

**Figure 1:**
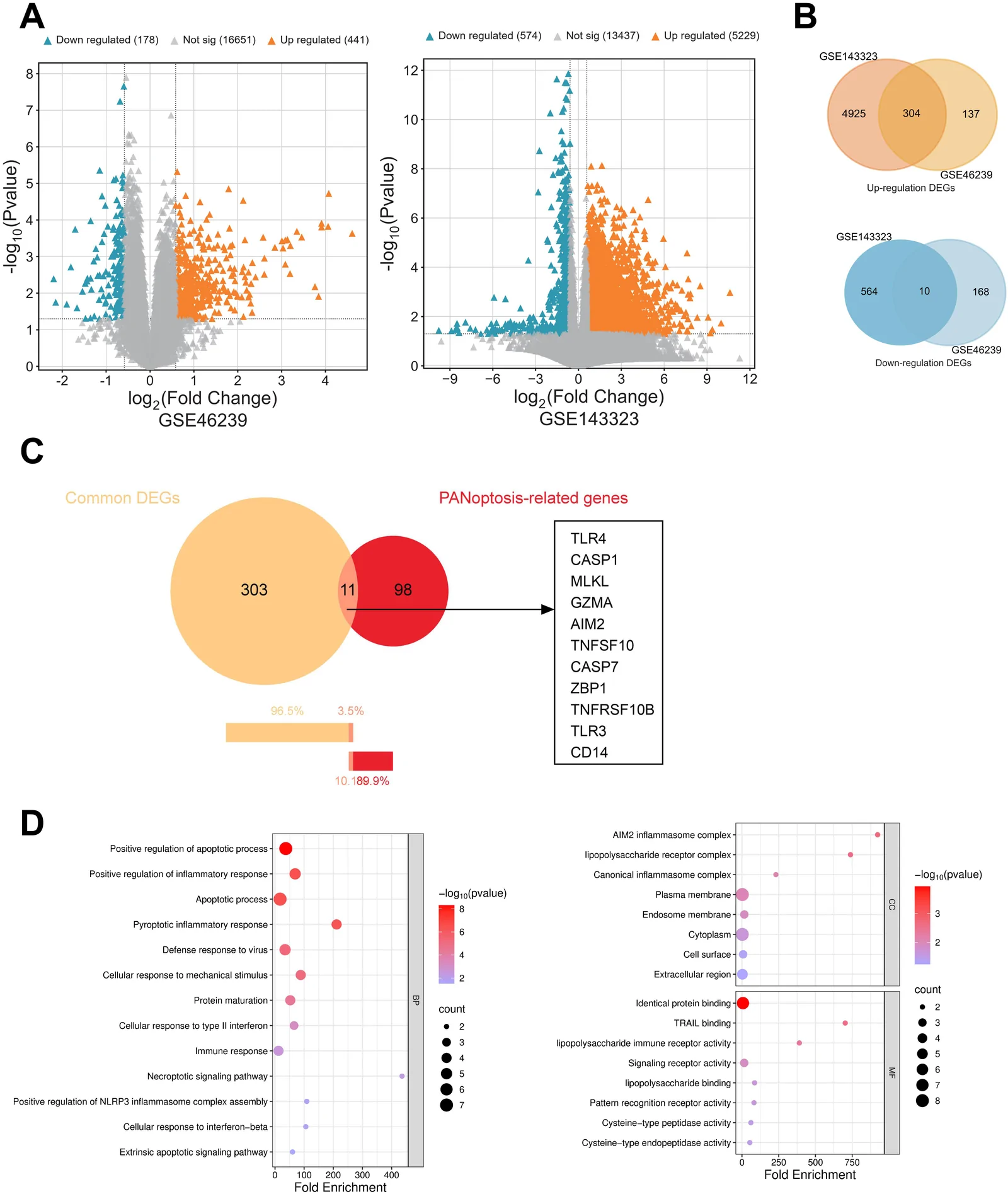
Bioinformatics identification of candidate PANoptosis-related genes in DM. **(A)** Volcano plots showing differentially expressed genes (DEGs) in the GSE46239 and GSE143323 datasets. **(B)** Venn diagram showing the overlap of DEGs between the GSE46239 and GSE143323 datasets. **(C)** Identification of candidate PANoptosis-related genes by intersecting the common DEGs with a curated set of PANoptosis-related genes. **(D)** Gene Ontology (GO) enrichment analysis of the candidate PANoptosis-related genes.

The shared differentially expressed genes were subsequently intersected with a curated set of PANoptosis-related genes, yielding 11 DM-associated candidate PANoptosis-related genes: *TLR4, CASP1, MLKL, GZMA, AIM2, TNFSF10, CASP7, ZBP1, TNFRSF10B, TLR3*, and *CD14* (**Figure 1C**). Functional enrichment analysis revealed that these candidate genes were primarily enriched in biological processes related to programmed cell death, including apoptosis, pyroptosis, and necroptosis. Enrichment was also observed in processes involving the cellular response to IFN-β, inflammatory responses, and immune regulation (**Figure 1D**). Collectively, these findings suggest that the identified PANoptosis-related genes may link dysregulated immune-inflammatory signaling to cell death-associated tissue injury in DM.

### 3.2 Machine learning-based identification of candidate core PANoptosis-related genes in dermatomyositis

The candidate gene set was further evaluated using three complementary machine-learning algorithms: LASSO regression, RF, and XGBoost. LASSO regression identified candidate features by selecting genes with nonzero coefficients at the optimal penalty parameter determined by cross-validation (**Figures 2A, B**). RF and XGBoost analyses ranked candidate genes according to their feature-importance scores (**Figures 2C, D**). The genes consistently selected by all three algorithms were integrated to identify robust candidate core PANoptosis-related genes (**Figure 2E**). Four core genes associated with DM were identified, including *ZBP1, CD14, TLR3*, and *TNFSF10*. These genes represent candidate molecular features associated with PANoptosis-related dysregulation in DM and were subsequently subjected to diagnostic evaluation and mechanistic investigation.

**Figure 2:**
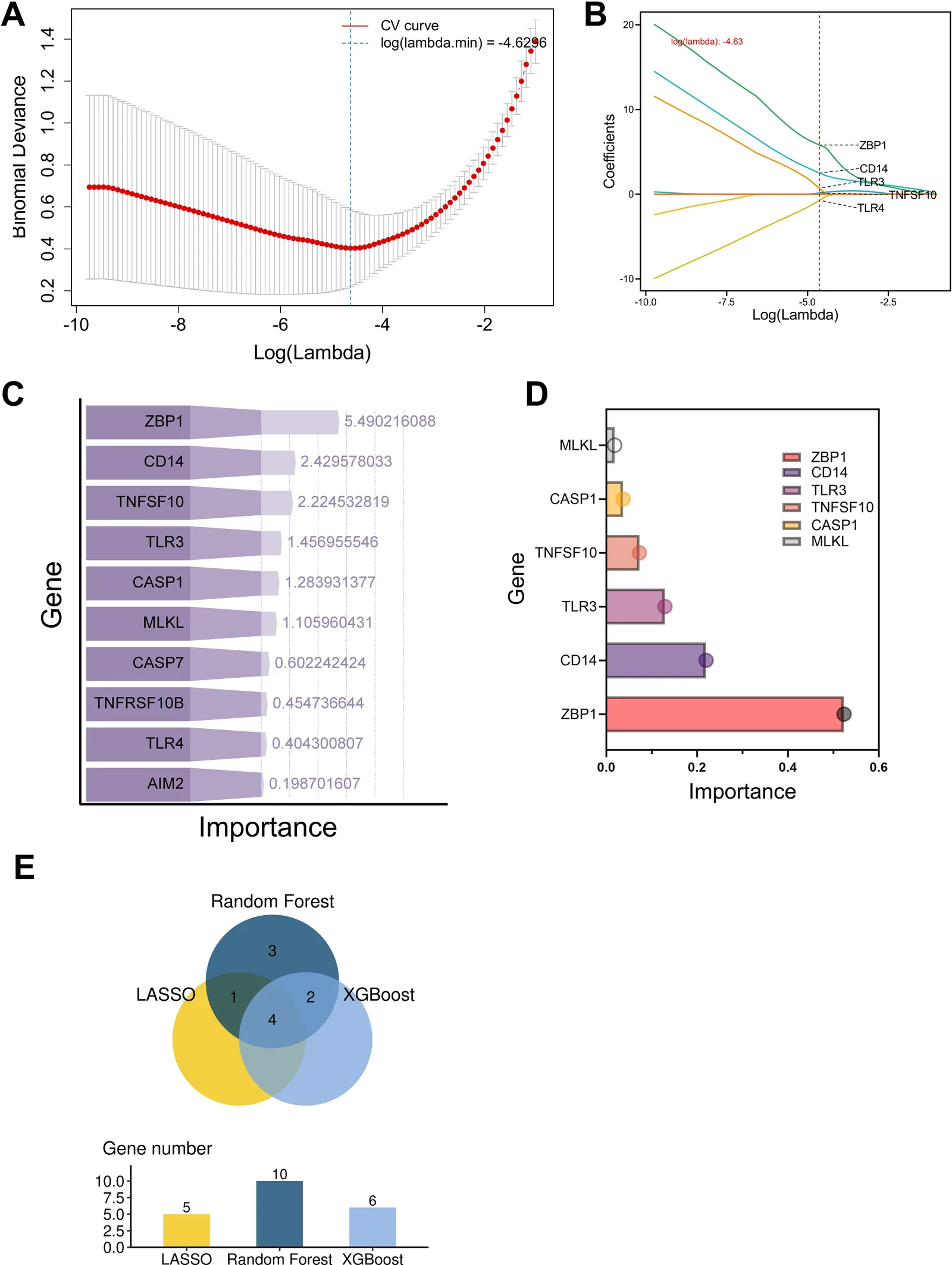
Machine learning-based identification of candidate core PANoptosis-related genes in DM. **(A, B)** Least absolute shrinkage and selection operator (LASSO) regression analysis of the candidate PANoptosis-related genes. **(C)** Feature-importance ranking generated using the random forest (RF) algorithm. **(D)** Top 10 genes ranked by feature importance using the extreme gradient boosting (XGBoost) algorithm. **(E)** Venn diagram showing the overlap among genes selected by LASSO, RF, and XGBoost.

### 3.3 Discriminatory performance of candidate core genes and development of a PANoptosis-related risk score

Receiver operating characteristic (ROC) curve analysis was used to assess the discriminatory performance of each of the four candidate core PANoptosis-related genes. *ZBP1, CD14, TLR3*, and *TNFSF10* yielded area under the ROC curve (AUC) values of 0.8333, 0.8958, 0.8490, and 0.8698, respectively, indicating their potential discriminatory value for DM (**Figure 3A**). The four genes were subsequently incorporated into a binary logistic regression model to generate a PANoptosis-related risk score (PRS). PRS values were significantly higher in DM samples than in control samples (**Figure 3B**). ROC curve analysis of the four-gene PRS yielded an AUC of 0.9375, with a 95% confidence interval (CI) of 0.8670–1.0000 (**Figure 3C**). The AUC of the combined PRS model was numerically higher than that of any individual gene, suggesting that integration of the four genes may provide the discrimination between DM and control samples.

**Figure 3:**
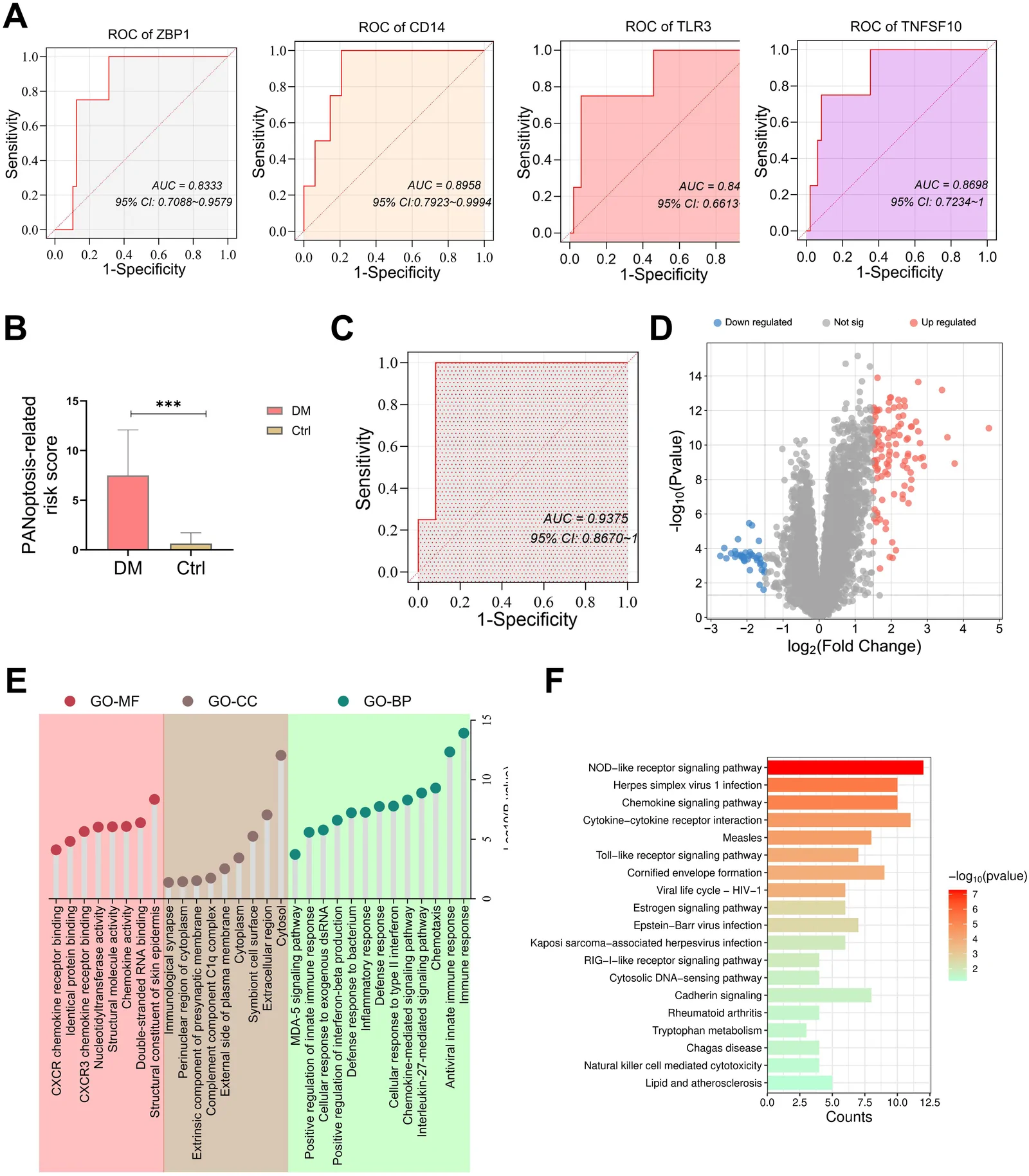
Construction of a PANoptosis-related risk score and evaluation of its discriminatory performance and transcriptomic stratification in DM. **(A)** Receiver operating characteristic (ROC) curves for the four candidate core PANoptosis-related genes. **(B)** Comparison of the PANoptosis-related risk score (PRS) between DM and control samples. **(C)** ROC curve of the four-gene PRS model. **(D)** Volcano plot showing differentially expressed genes (DEGs) between the high- and low-PRS groups, stratified according to the median PRS value. **(E)** Gene Ontology (GO) enrichment analysis of DEGs between the high- and low-PRS groups. **(F)** Kyoto Encyclopedia of Genes and Genomes (KEGG) pathway enrichment analysis of DEGs between the high- and low-PRS groups. ***P<0.001.

Samples were then stratified into high- and low-PRS groups according to the median PANoptosis-related risk score (PRS) to examine whether PRS-based stratification captured broader transcriptomic differences in DM. Differential expression analysis between the two groups identified 146 differentially expressed genes (DEGs), of which 109 were upregulated and 37 were downregulated in the high-PRS group relative to the low-PRS group (**Figure 3D**). Gene Ontology (GO) enrichment analysis identified terms related to immune responses, melanoma differentiation-associated protein 5 signaling, cellular responses to exogenous double-stranded RNA, and the complement C1q complex (**Figure 3E**). Kyoto Encyclopedia of Genes and Genomes (KEGG) pathway analysis further revealed significant enrichment of innate immune pathways, including the NOD-like receptor signaling pathway, Toll-like receptor signaling pathway, RIG-I-like receptor signaling pathway, and cytosolic DNA-sensing pathway. Enrichment was also observed in cytokine-cytokine receptor interaction, chemokine signaling, and natural killer cell-mediated cytotoxicity pathways (**Figure 3F**). Collectively, these findings indicate that PRS-based stratification captures distinct transcriptional profiles associated with innate immune and inflammatory signaling in DM.

### 3.4 Altered immune cell composition in dermatomyositis skeletal muscle

The immune landscape of DM skeletal muscle was characterized by applying CIBERSORTx with the leukocyte signature matrix 22 (LM22) to the GSE143323 expression dataset. The inferred relative abundance of the 22 immune cell subsets differed markedly between DM and control samples (**Figures 4A, B**). These findings indicate broad alterations in the immune cell composition of DM skeletal muscle. Spearman correlation analysis was subsequently performed to examine the relationships between the expression levels of the four candidate core PANoptosis-related genes and the inferred abundance of immune cell subsets. The expression levels of *ZBP1, CD14, TLR3*, and *TNFSF10* were positively correlated with the estimated abundance of M1 macrophages. In addition, *CD14* and *ZBP1* expression correlated positively with memory B cells and γδ T cells, whereas *TLR3* and *TNFSF10* expression correlated positively with T follicular helper cells (**Figure 4C**). Collectively, these findings link the four candidate core genes to distinct myeloid and adaptive immune cell profiles in DM skeletal muscle, suggesting a potential relationship between PANoptosis-related transcriptional dysregulation and the altered immune microenvironment.

**Figure 4:**
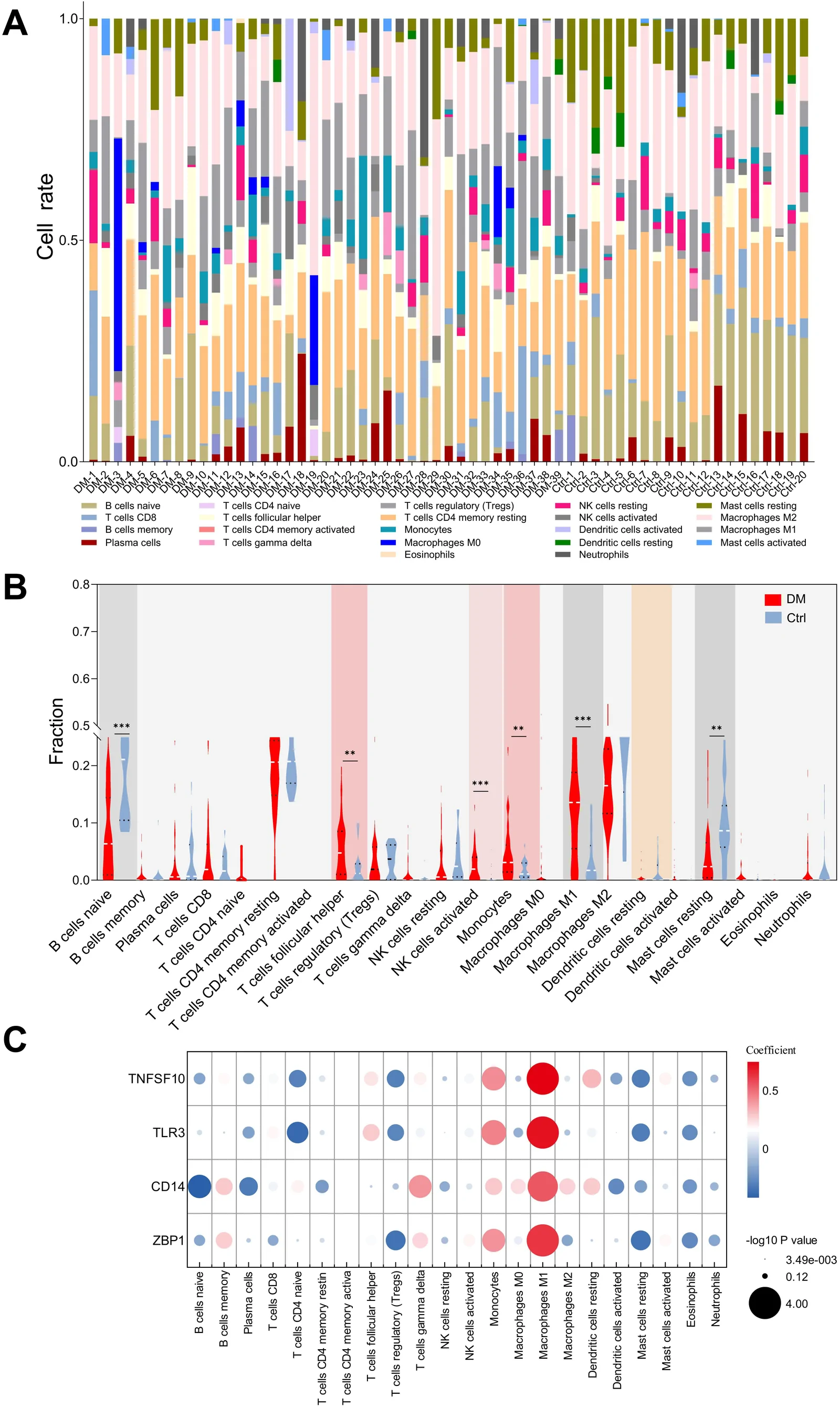
Immune cell composition in DM skeletal muscle and its association with candidate core PANoptosis-related genes. **(A)** Heatmap showing the inferred relative abundance of 22 immune cell subsets estimated using CIBERSORTx with the leukocyte signature matrix 22 (LM22). **(B)** Comparison of the inferred abundance of the 22 immune cell subsets between DM and control (Ctrl) samples. **(C)** Correlations between the expression levels of the four candidate core PANoptosis-related genes, *ZBP1, CD14, TLR3*, and *TNFSF10*, and the inferred abundance of the 22 immune cell subsets. **P < 0.01, ***P < 0.001.

### 3.5 Clinical validation of candidate core PANoptosis-related genes and their associations with serum markers of muscle injury

PBMCs were obtained from 25 patients with DM and 15 HCs. RT-qPCR analysis showed that the mRNA levels of all four candidate core genes, *ZBP1, CD14, TLR3*, and *TNFSF10*, were significantly higher in patients with DM than in HCs (**Figure 5A**). These results confirmed the upregulation of the four-gene signature in PBMCs from patients with DM.

**Figure 5:**
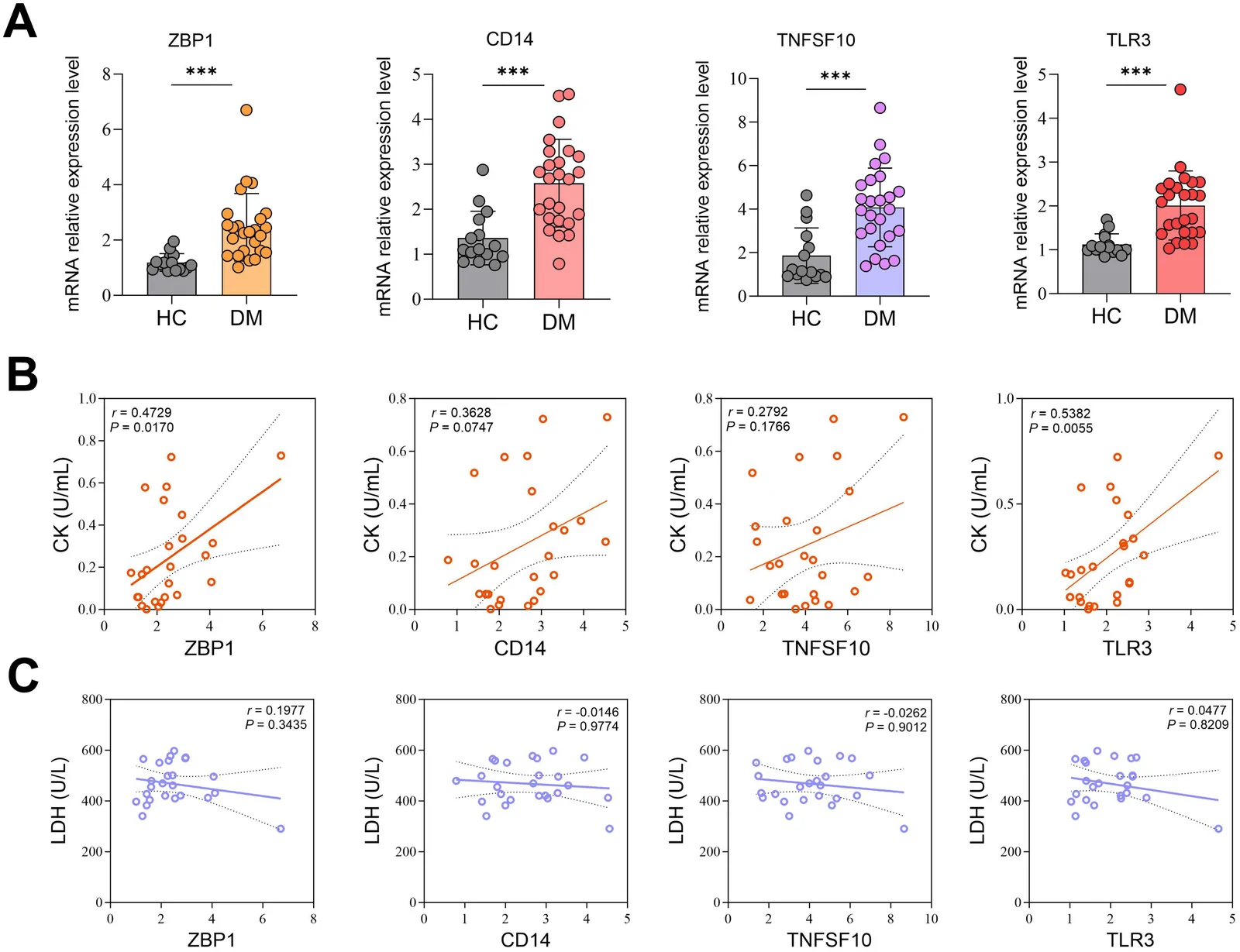
Expression of candidate core PANoptosis-related genes in peripheral blood mononuclear cells from patients with dermatomyositis and their associations with serum markers of muscle injury. **(A)** Relative mRNA expression levels of *ZBP1, CD14, TLR3*, and *TNFSF10* in peripheral blood mononuclear cells (PBMCs) from patients with dermatomyositis (DM) and healthy controls (HCs). **(B)** Correlations between the expression levels of the four candidate core genes and serum creatine kinase (CK) levels in patients with DM. **(C)** Correlations between the expression levels of the four candidate core genes and serum lactate dehydrogenase (LDH) levels in patients with DM. ***P < 0.001.

Spearman correlation analysis further revealed that ZBP1 and TLR3 mRNA levels were positively correlated with serum creatine kinase (CK) levels, whereas neither gene showed a significant correlation with serum lactate dehydrogenase (LDH) levels (**Figures 5B, C**). These associations link elevated ZBP1 and TLR3 expression to biochemical evidence of muscle injury, although they do not establish a direct relationship with clinical disease severity. Given its consistent prioritization across the bioinformatics, immune cell deconvolution, and clinical validation analyses, together with its established relevance to PANoptosis-related signaling, ZBP1 was selected for subsequent functional and mechanistic studies.

### 3.6 IFN-β induces PANoptosis-like injury in adult human skeletal muscle cells

The CCK-8 assay showed that cell viability was significantly lower in the IFN-β-treated group than in the untreated control group (Ctrl) (**Figure 6A**). Consistently, LDH release into the culture supernatant was significantly increased following IFN-β treatment, indicating loss of plasma membrane integrity and increased HSkMC injury (**Figure 6B**). Transmission electron microscopy revealed well-preserved cellular ultrastructure in control HSkMCs, characterized by continuous plasma membranes, clearly defined mitochondrial cristae, and evenly distributed chromatin. In contrast, IFN-β-treated cells displayed heterogeneous ultrastructural abnormalities associated with multiple forms of programmed cell death. Some cells exhibited chromatin margination and apoptotic body formation, consistent with apoptotic injury. Other cells showed cellular swelling, plasma membrane disruption, and release of cytoplasmic contents, features compatible with pyroptosis-like injury. Severe mitochondrial swelling, disruption of mitochondrial cristae, cytoplasmic vacuolization, and cellular disintegration were also observed, consistent with necroptosis-like damage. Collectively, IFN-β-treated HSkMCs exhibited concurrent ultrastructural features associated with apoptosis, pyroptosis, and necroptosis (**Figure 6C**).

**Figure 6:**
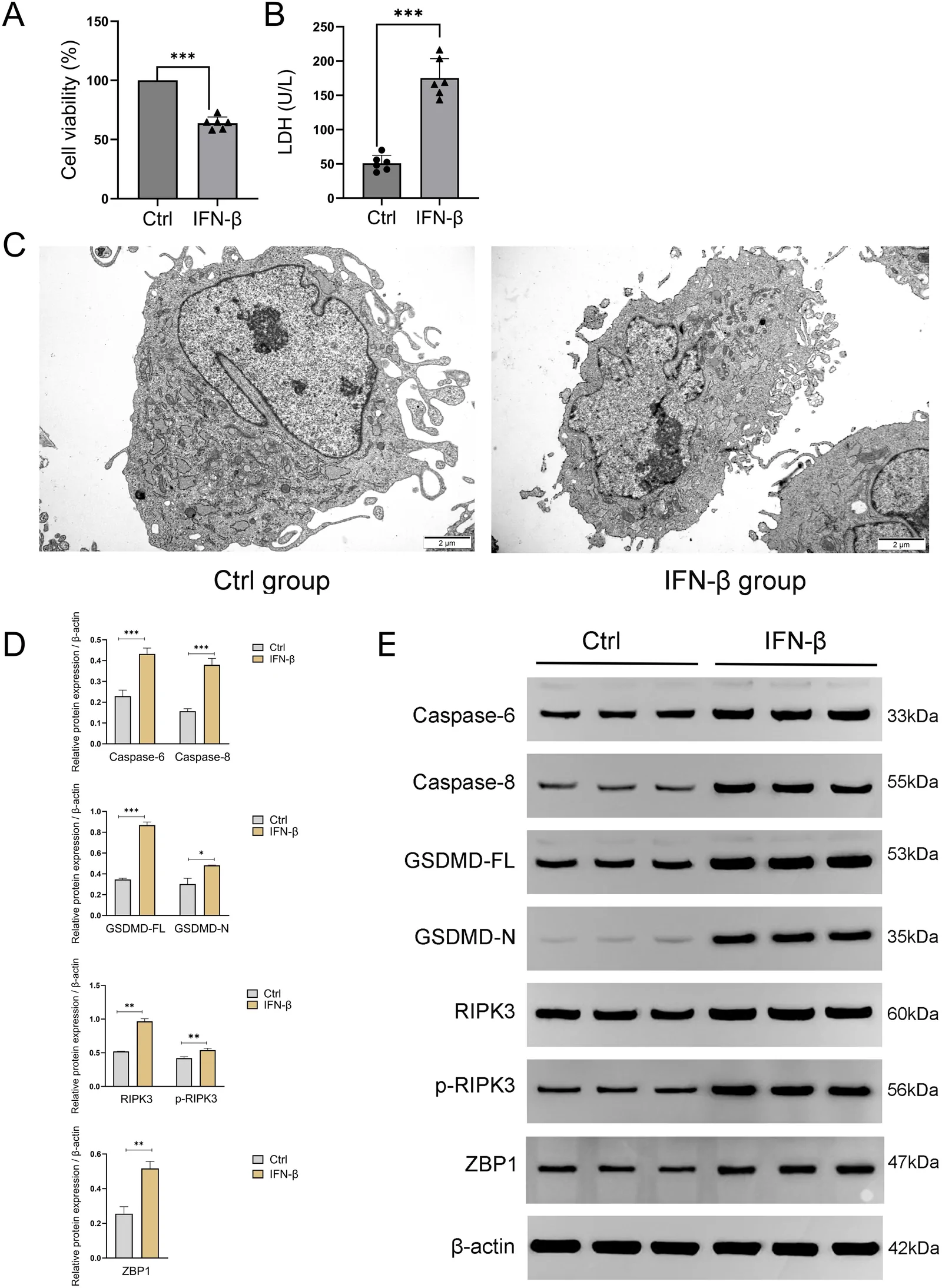
Interferon-β induces PANoptosis-like injury in adult human skeletal muscle cells. **(A)** Cell viability of adult human skeletal muscle cells (HSkMCs) in the control (Ctrl) and IFN-β-treated groups, as assessed using the Cell Counting Kit-8 (CCK-8) assay. **(B)** Lactate dehydrogenase (LDH) release into the culture supernatant in the Ctrl and IFN-β-treated groups. **(C)** Representative transmission electron microscopy images showing ultrastructural changes in HSkMCs following IFN-β treatment. **(D, E)** Representative western blot images and densitometric quantification of ZBP1, caspase-8, caspase-6, full-length gasdermin D (GSDMD-FL), the N-terminal fragment of gasdermin D (GSDMD-N), receptor-interacting serine/threonine-protein kinase 3 (RIPK3), and phosphorylated RIPK3 (p-RIPK3) in the Ctrl and IFN-β-treated groups. *P < 0.05, **P < 0.01, ***P < 0.001.

Western blotting showed that IFN-β treatment significantly increased the protein levels of ZBP1, caspase-8, caspase-6, full-length gasdermin D (GSDMD-FL), and receptor-interacting serine/threonine-protein kinase 3 (RIPK3). The levels of the N-terminal fragment of gasdermin D (GSDMD-N) and phosphorylated RIPK3 (p-RIPK3) were also significantly elevated (**Figures 6D, E**). Together, the ultrastructural and molecular findings support the induction of PANoptosis-like injury and enhanced PANoptosis-associated signaling in IFN-β-treated HSkMCs.

### 3.7 ZBP1 contributes to IFN-β-induced PANoptosis-associated signaling in adult human skeletal muscle cells

Given the PANoptosis-like changes observed in IFN-β-treated HSkMCs, we next investigated the involvement of ZBP1 in this process. Based on its consistent prioritization in the bioinformatics analyses and clinical validation, ZBP1 expression was genetically manipulated in HSkMCs using ZBP1-targeting siRNAs and a ZBP1 overexpression plasmid. The knockdown efficiencies of three ZBP1-targeting siRNAs were first evaluated by RT-qPCR. Compared with the negative-control siRNA (si-NC), si1-ZBP1, si2-ZBP1, and si3-ZBP1 each significantly reduced ZBP1 mRNA expression. Among the three sequences, si2-ZBP1 produced the greatest reduction in ZBP1 mRNA levels and was therefore selected for subsequent experiments (**Figure 7A**). Conversely, ZBP1 mRNA expression was significantly higher in cells transfected with pcDNA3.1-ZBP1 than in those transfected with the corresponding empty vector, confirming effective ZBP1 overexpression (**Figure 7B**). Western blotting further showed that si2-ZBP1 markedly reduced ZBP1 protein levels, whereas pcDNA3.1-ZBP1 significantly increased ZBP1 protein expression. Together, these results confirmed the effective establishment of ZBP1 knockdown and overexpression systems in HSkMCs (**Figures 7C, D**).

**Figure 7:**
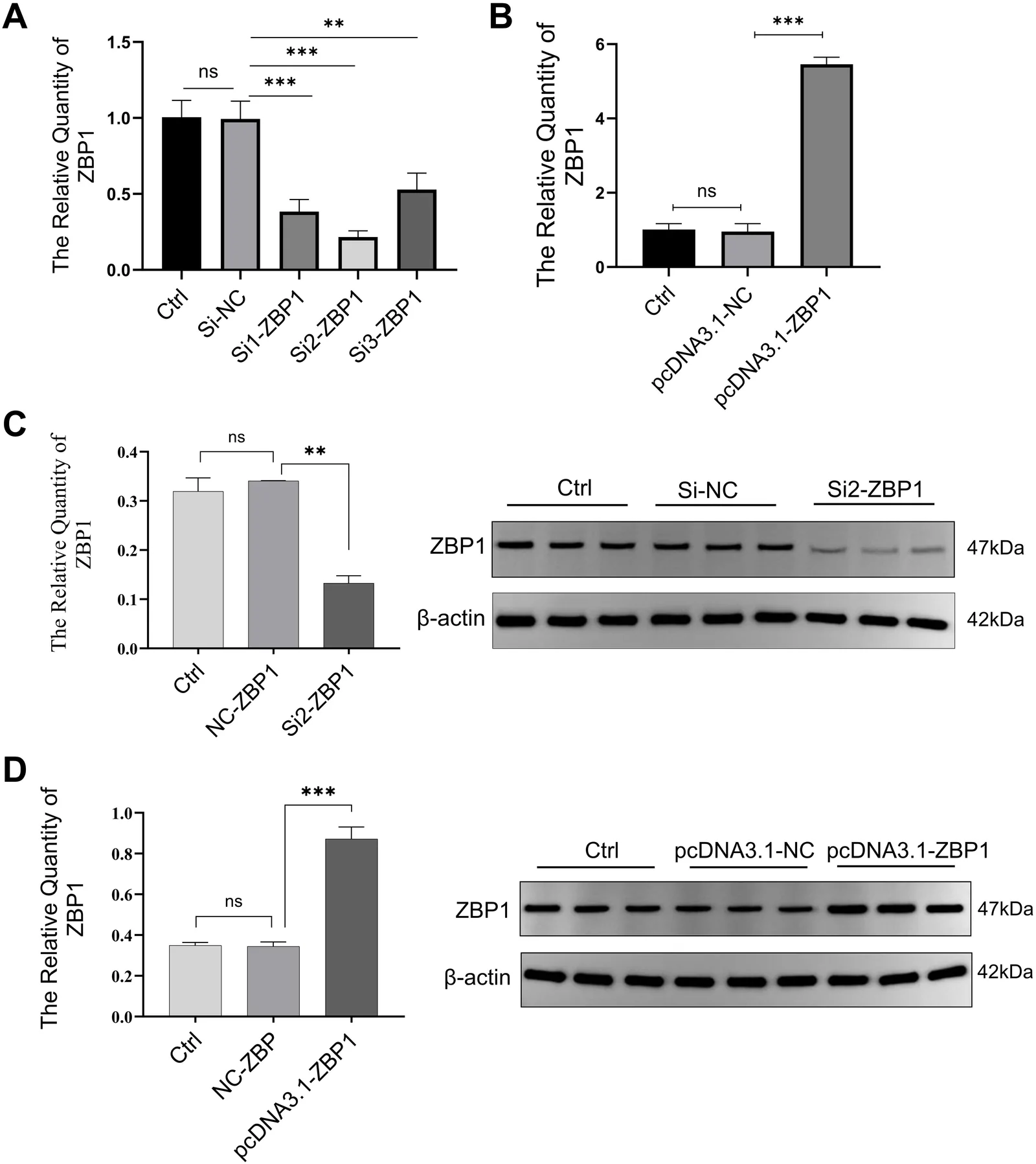
Establishment and validation of ZBP1 knockdown and overexpression systems in HSkMCs. **(A)** Reverse transcription quantitative polymerase chain reaction (RT-qPCR) analysis of ZBP1 mRNA expression following transfection with three ZBP1-targeting small interfering RNAs (siRNAs). si2-ZBP1 exhibited the greatest knockdown efficiency and was selected for subsequent experiments. **(B)** RT-qPCR analysis of ZBP1 mRNA expression following transfection with the ZBP1 overexpression plasmid or the corresponding empty vector. **(C, D)** Representative western blot images and densitometric quantification of ZBP1 protein expression following ZBP1 knockdown and overexpression. **P < 0.01, ***P < 0.001; ns, not significant.

To specifically assess whether increased ZBP1 abundance was associated with enhanced recruitment of PANoptosis-related proteins, Co-IP analysis was performed in HSkMCs following ZBP1 overexpression in the absence of IFN-β stimulation. Co-IP analysis showed that ZBP1 overexpression increased the co-precipitation of RIPK3, caspase-8, and caspase-6 with ZBP1, supporting enhanced association of ZBP1 with these PANoptosis-related signaling proteins (**Figures 8A, B**). We next examined the effects of ZBP1 knockdown on IFN-β-induced PANoptosis-associated signaling. Compared with the IFN-β + si-NC group, the IFN-β + si-ZBP1 group showed significantly lower protein levels of caspase-8, caspase-6, RIPK3, GSDMD-FL, and GSDMD-N. p-RIPK3 levels were also markedly reduced following ZBP1 knockdown (**Figures 8C, D**). These findings indicate that ZBP1 knockdown attenuates IFN-β-induced PANoptosis-associated molecular changes in HSkMCs.

**Figure 8:**
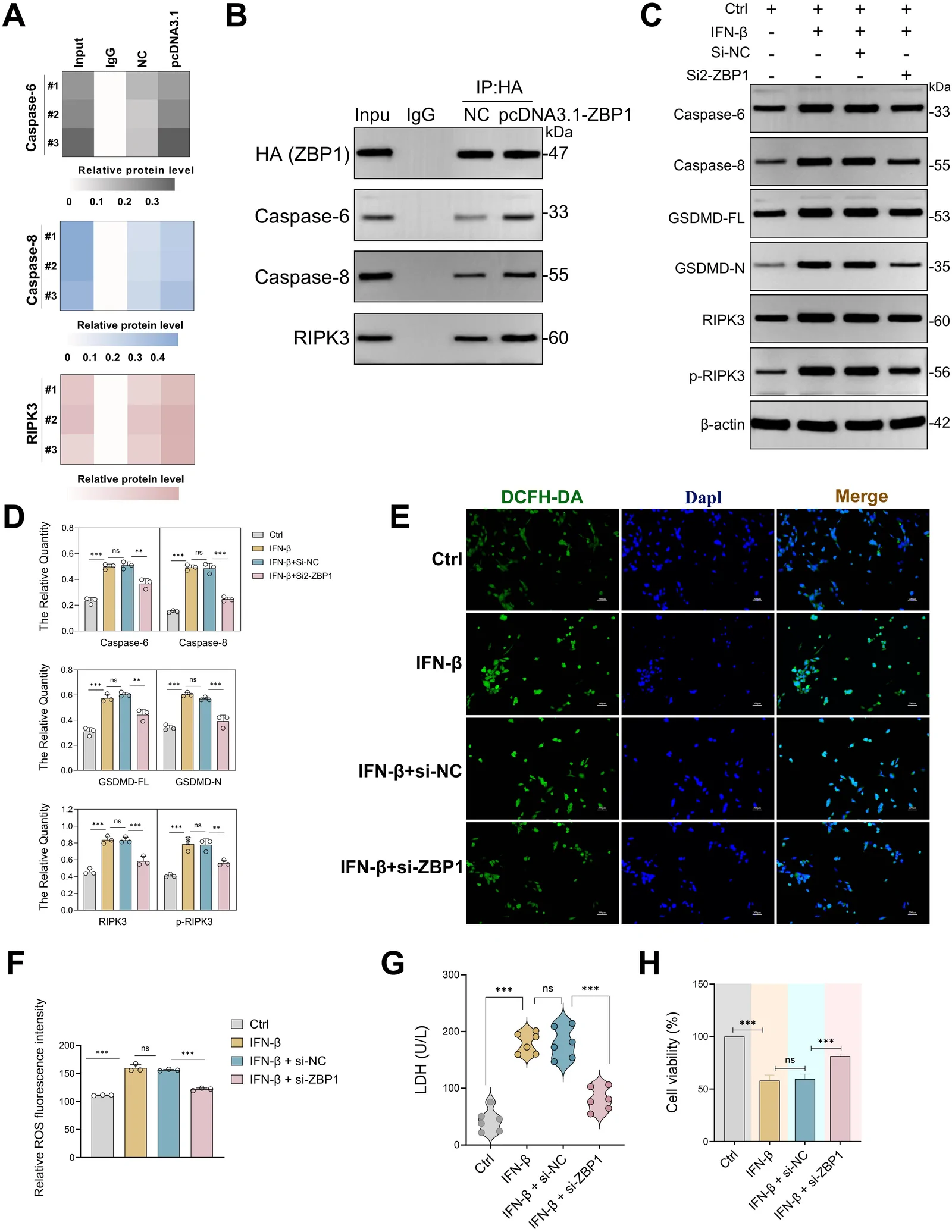
ZBP1 contributes to IFN-β-induced PANoptosis-associated signaling and cellular injury in HSkMCs. **(A)** Representative co-immunoprecipitation images showing the association of ZBP1 with RIPK3, caspase-8, and caspase-6. **(B)** Densitometric quantification of RIPK3, caspase-8, and caspase-6 co-precipitated with ZBP1. **(C, D)** Representative western blot images and densitometric quantification of ZBP1, caspase-8, caspase-6, full-length gasdermin D (GSDMD-FL), the N-terminal fragment of gasdermin D (GSDMD-N), RIPK3, and phosphorylated RIPK3 (p-RIPK3) following ZBP1 knockdown in IFN-β-treated HSkMCs. **(E)** Representative fluorescence images showing intracellular reactive oxygen species (ROS) accumulation following ZBP1 knockdown. **(F)** Quantification of ROS fluorescence intensity. **(G)** Cell viability assessed using the Cell Counting Kit-8 (CCK-8) assay. **(H)** Lactate dehydrogenase (LDH) release into the culture supernatant. **P < 0.01, ***P < 0.001, ns, not significant.

### 3.8 Baicalin binds ZBP1 and may attenuate PANoptosis-associated injury in adult human skeletal muscle cells

Given the absence of a high-resolution experimental structure covering the ZBP1 RHIM regions, the AlphaFold-predicted full-length human ZBP1 structure was subjected to unbiased binding-pocket prediction using CB-Dock2, which identified five candidate pockets (**Figure 9A**). The extent of residue overlap between each predicted pocket and the RHIM regions was then evaluated. Site 1 exhibited the greatest overlap with RHIM-associated residues and was therefore selected for subsequent molecular docking with baicalin. Molecular docking of baicalin to Site 1 of ZBP1 generated nine predicted binding poses. Among them, pose 1 exhibited the lowest predicted binding energy (−9.172 kcal/mol) and showed multiple noncovalent interactions with residues assigned to RHIM1 and RHIM2. Specifically, baicalin was predicted to form hydrogen bonds with Asn195, Ser196, and Asp253, a salt bridge with Arg216, π-related and hydrophobic interactions with Trp197, and a hydrophobic contact with Met272 (**Figure 9B**). These findings further support the possibility that baicalin may occupy a predicted binding pocket that overlaps the RHIM-containing regions of ZBP1. MST was subsequently performed to experimentally evaluate the binding of baicalin to ZBP1. MST confirmed direct binding between baicalin and ZBP1, with an equilibrium dissociation constant (K_D_) of 7.12 μM (**Figure 9C**), indicating micromolar binding affinity.

**Figure 9:**
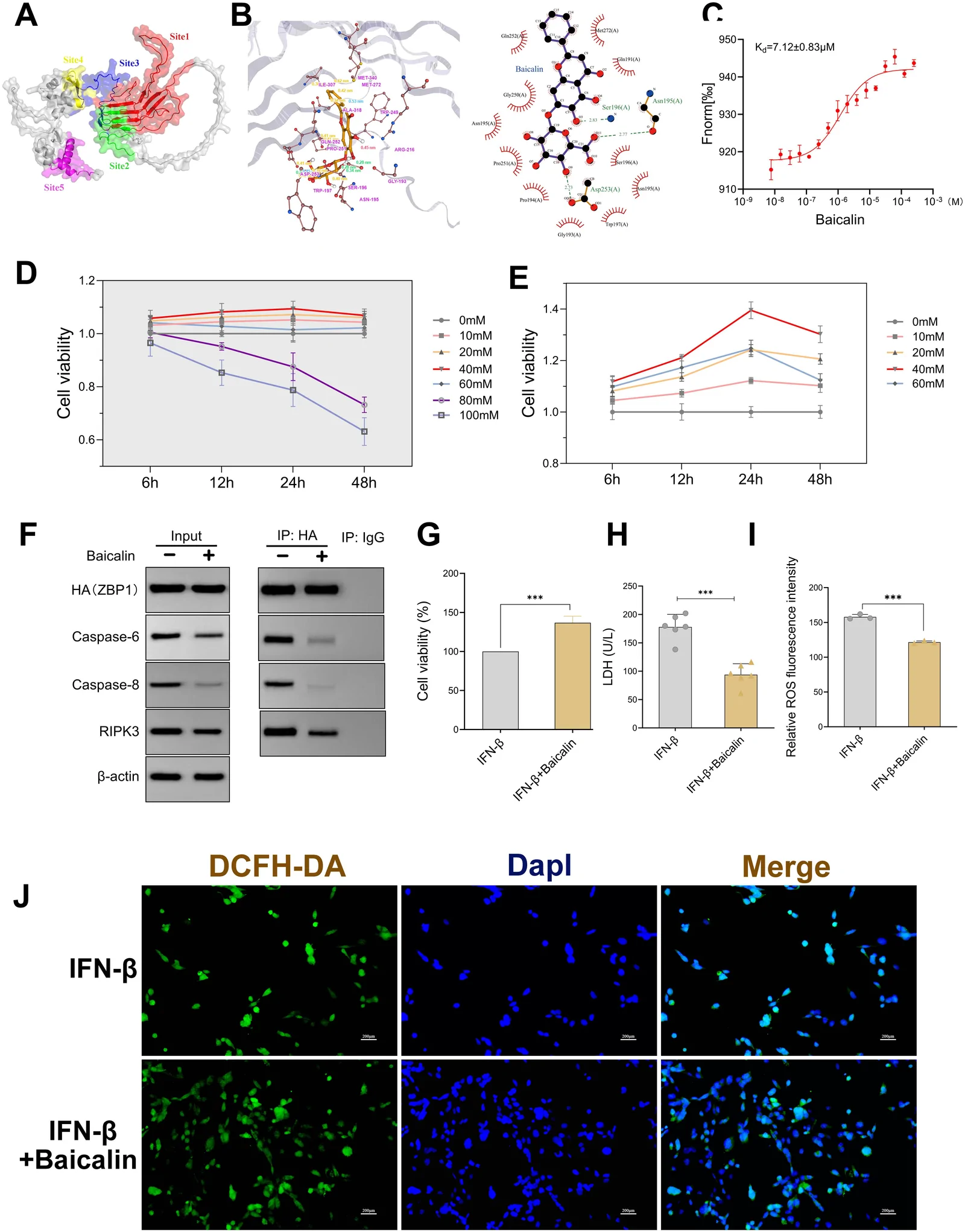
Baicalin binds ZBP1 and attenuates IFN-β-induced PANoptosis-associated injury in HSkMCs. **(A)** Potential druggable binding pockets identified from the predicted full-length human Z-DNA-binding protein 1 (ZBP1) structure (AlphaFold ID: AF-Q9H171-F1). **(B)** Two-dimensional and three-dimensional interaction patterns of the predicted binding conformation between Baicalin and human ZBP1 Site 1. **(C)** Fitted microscale thermophoresis (MST) binding curve for baicalin and ZBP1. **(D)** Viability of untreated HSkMCs following exposure to different concentrations of baicalin for the indicated durations, used to identify a non-cytotoxic concentration range. **(E)** Effects of different baicalin concentrations and treatment durations on the viability of IFN-β-treated HSkMCs. **(F)** Representative co-immunoprecipitation images showing the association of ZBP1 with receptor-interacting serine/threonine-protein kinase 3 (RIPK3), caspase-8, and caspase-6 following baicalin treatment. **(G)** Viability of IFN-β-treated HSkMCs following baicalin treatment, as assessed using the Cell Counting Kit-8 (CCK-8) assay. **(H)** Lactate dehydrogenase (LDH) release into the culture supernatant. **(I)** Quantification of intracellular reactive oxygen species (ROS) fluorescence intensity. **(J)** Representative fluorescence images showing intracellular ROS accumulation in each group. ***P < 0.001.

Safety profiling showed that baicalin at concentrations of 10–60 μM did not significantly affect the viability of untreated HSkMCs at any of the assessed time points. By contrast, exposure to 80 or 100 μM baicalin progressively reduced cell viability with increasing treatment duration, indicating concentration- and time-dependent cytotoxicity at higher concentrations (**Figure 9D**). The concentration- and time-dependent effects of baicalin were subsequently evaluated in the IFN-β-induced HSkMC injury model. Within the non-cytotoxic concentration range, baicalin increased the viability of IFN-β-treated HSkMCs, with the greatest protective effect observed following treatment with 40 μM baicalin for 24 h. Increasing the concentration to 60 μM or extending the treatment duration to 48 h conferred no additional improvement in cell viability. Based on its safety profile and protective effect, treatment with 40 μM baicalin for 24 h was selected for subsequent functional and mechanistic experiments (**Figure 9E**).

Co-IP analysis was subsequently performed to assess the effects of baicalin on ZBP1-associated protein complexes. Baicalin treatment reduced the co-precipitation of RIPK3, caspase-8, and caspase-6 with ZBP1 in HSkMCs (**Figure 9F**). These findings suggest that baicalin attenuates the association of ZBP1 with multiple PANoptosis-related proteins and may thereby limit the formation of ZBP1-associated cell death signaling complexes. Functional assays further showed that baicalin alleviated IFN-β-induced injury in HSkMCs. The CCK-8 assay demonstrated that baicalin significantly restored cell viability following IFN-β exposure (**Figure 9G**). Consistently, baicalin reduced LDH release into the culture supernatant (**Figure 9H**) and markedly decreased intracellular ROS accumulation (**Figures 9I, J**). Collectively, these results indicate that baicalin improves cell viability and attenuates plasma membrane injury and oxidative stress in IFN-β-treated HSkMCs.

## 4 Discussion

Previous studies of inflammatory myopathies have largely examined apoptosis, pyroptosis, and necroptosis as separate processes. Necroptosis-associated signaling involving receptor-interacting serine/threonine-protein kinase 3 (RIPK3) and mixed lineage kinase domain-like pseudokinase (MLKL) has been detected in skeletal muscle fibers from patients with idiopathic inflammatory myopathies and associated with myofiber injury and local inflammatory responses (19, 33, 34). In parallel, pyruvate kinase M2 (PKM2)-driven glycolytic reprogramming has been reported to promote NOD-like receptor family pyrin domain-containing 3 (NLRP3) inflammasome-dependent pyroptosis in skeletal muscle cells in dermatomyositis and polymyositis (DM/PM) (18, 35). Collectively, these observations support the involvement of multiple regulated cell death pathways in skeletal muscle injury in DM.

Nevertheless, interpreting these pathways independently may not adequately explain the co-occurrence of plasma membrane disruption, inflammasome-related signaling, caspase activation, and RIPK3 phosphorylation within affected skeletal muscle (36). PANoptosis instead describes an integrated cell death program characterized by extensive crosstalk among the molecular machinery of pyroptosis, apoptosis, and necroptosis, rather than the mere simultaneous presence of three independent death pathways. This concept may therefore provide a useful integrative framework for understanding the coordinated engagement of multiple cell death pathways and the amplification of inflammatory injury in DM (37, 38). Within this framework, our findings of concurrent ultrastructural abnormalities and coordinated changes in ZBP1, GSDMD-N, caspase-8, caspase-6, and p-RIPK3 support the presence of PANoptosis-like injury and PANoptosis-associated signaling in IFN-β-treated HSkMCs.

By integrating concordantly expressed genes shared between DM skin and skeletal muscle with a curated set of 109 PANoptosis-related genes, we identified 11 candidate genes: *TLR4, CASP1, MLKL, GZMA, AIM2, TNFSF10, CASP7, ZBP1, TNFRSF10B, TLR3*, and *CD14*. These genes participate in several interconnected processes, including innate immune sensing, inflammasome signaling, death receptor pathways, caspase-mediated signaling, and the execution of necroptosis. Their enrichment in cell death-, inflammation-, and immune regulation-related processes further supports the potential involvement of PANoptosis-associated molecular networks in DM. Despite the distinct cellular and immunological environments of the skin and skeletal muscle, the shared transcriptional signature suggests that these tissues may exhibit overlapping abnormalities in immune sensing and regulated cell death.

Consensus feature selection using LASSO regression, RF, and XGBoost prioritized ZBP1, CD14, TLR3, and TNFSF10 as candidate core genes. Each gene showed discriminatory ability between DM and control samples, with area under the curve values ranging from 0.8333 to 0.8958. The four-gene PRS was significantly elevated in DM and achieved an area under the curve of 0.9375, suggesting that the combined signature may better capture coordinated alterations in immune-inflammatory and regulated cell-death pathways than individual genes. Consistently, PRS-based stratification revealed broader transcriptional differences, with enrichment in innate immune sensing pathways, including MDA5, NOD-like receptor, Toll-like receptor, and cytosolic DNA-sensing signaling, as well as cytokine, chemokine, and natural killer cell-mediated cytotoxicity pathways. These findings suggest that the PRS reflects a broader immune-inflammatory transcriptional state in DM. However, because it was derived from cross-sectional case-control transcriptomic data, the PRS should be regarded as a discriminatory molecular score rather than a predictor of future clinical risk.

Among the four candidate core genes, ZBP1 was prioritized for further investigation. Clinical validation showed that *ZBP1* mRNA expression was elevated in PBMCs from patients with DM and positively correlated with serum CK levels. This association links increased peripheral ZBP1 expression to skeletal muscle injury. ZBP1 is an interferon-inducible cytosolic nucleic acid sensor that recognizes Z-DNA and Z-RNA through its Zα domains and engages RHIM-containing signaling proteins, particularly RIPK3, through its RHIM (39–41). Previous studies have shown that ZBP1 can promote RIPK3-dependent necroptosis and, in specific cellular contexts, coordinate pyroptotic, apoptotic, and necroptotic signaling through the assembly of multiprotein death complexes involving caspase-8 and inflammasome-associated components (42, 43). Elevated ZBP1 may represent a molecular feature associated with immune-inflammatory activity and muscle injury in DM. However, because PBMC gene expression may also be influenced by treatment exposure, systemic inflammatory status, and differences in immune-cell composition, the present findings do not establish ZBP1 as a DM-specific biomarker.

Although ZBP1 is classically activated by Z-form nucleic acids, accumulating evidence indicates that its cell death-promoting activity can also occur under conditions not strictly dependent on canonical Z-nucleic acid sensing. Zhang et al. reported that forced ZBP1 overexpression was sufficient to induce PANoptosis in cardiomyocytes both *in vitro* and *in vivo*, indicating that, at least under these experimental conditions, sufficiently high ZBP1 abundance may favor the assembly of cell death signaling complexes even in the absence of a defined upstream ligand (44). Similarly, a study of heatstroke-associated injury showed that ZBP1-mediated cell death was independent of Z-form nucleic acid sensing but remained dependent on its RHIM (45). Ye et al. further demonstrated that, under sterile conditions, mitochondrial ROS promoted ZBP1 activation and aggregation through a mechanism that did not require its Zα domains (46). These findings suggest that ZBP1 activity may also be influenced by protein abundance, oxidative stress, and the intracellular microenvironment. Consistent with this concept, a recent study demonstrated IFN-β-induced ZBP1-dependent PANoptosis in T cells from patients with anti-MDA5-positive DM (47). Our findings extend this observation by suggesting that an IFN-β–ZBP1 axis may also contribute to PANoptosis-like injury in HSkMCs, supporting ZBP1 as a potential inflammatory cell death-associated node linking peripheral immune dysregulation with skeletal muscle injury in DM.

In the present study, IFN-β was used to model a type I interferon-rich environment relevant to DM muscle injury. IFN-β reduced HSkMC viability, increased LDH release and ROS accumulation, and induced ultrastructural features consistent with apoptosis-, pyroptosis-, and necroptosis-like injury. These changes were accompanied by increased GSDMD-N and p-RIPK3 levels, together with elevated ZBP1, caspase-8, and caspase-6, supporting activation of PANoptosis-related signaling. In infectious settings, IFN-β has been reported to induce ZBP1-dependent PANoptosis, which may counteract otherwise protective interferon responses (48). Based on our findings and previous evidence, IFN-β-induced PANoptosis-like injury in HSkMCs may involve two interconnected events. First, IFN-β may increase ZBP1 abundance, thereby priming skeletal muscle cells for inflammatory cell death signaling. Subsequently, oxidative stress, mitochondrial dysfunction, or altered conformations of endogenous nucleic acids may provide additional signals that promote ZBP1 activation and the assembly of downstream cell death complexes (49, 50). This proposed model remains inferential and requires direct temporal and mechanistic validation.

Nevertheless, the protein association and genetic perturbation experiments further support the involvement of ZBP1 in IFN-β-induced injury. ZBP1 overexpression increased the co-precipitation of RIPK3, caspase-8, and caspase-6 with ZBP1, whereas ZBP1 knockdown reduced the protein levels of caspase-8, caspase-6, RIPK3, GSDMD-FL, GSDMD-N, and p-RIPK3. Functionally, ZBP1 depletion also decreased intracellular ROS accumulation and LDH release while improving cell viability. RIPK3 and caspase-8 are central regulators of necroptotic and apoptotic signaling, respectively, whereas GSDMD-N functions as a pore-forming execution fragment of pyroptosis (51). Caspase-8 also participates in the crosstalk between apoptotic and necroptotic pathways. In addition, previous studies have shown that caspase-6 can promote RHIM-dependent signaling between ZBP1 and RIPK3, thereby amplifying ZBP1-driven inflammatory cell death (52, 53). Collectively, our findings suggest that ZBP1 contributes to IFN-β-induced PANoptosis-associated injury by facilitating the coordinated engagement of RIPK3-, caspase-, and GSDMD-related signaling.

Baicalin is a major bioactive flavonoid derived from Scutellaria baicalensis Georgi and has well-documented anti-inflammatory, antioxidant, and immunomodulatory properties. It has also been implicated in the regulation of multiple forms of regulated cell death (54–56). In the present study, molecular docking predicted that baicalin could occupy a candidate pocket in ZBP1 that may be relevant to RHIM-mediated protein associations. Microscale thermophoresis further confirmed direct binding between baicalin and ZBP1, with a K_D_ value of 7.12 μM. Moreover, Co-IP analysis showed that baicalin treatment reduced the co-precipitation of RIPK3, caspase-8, and caspase-6 with ZBP1, supporting the possibility that baicalin limits the formation of ZBP1-associated cell death signaling complexes. Functionally, baicalin improved cell viability while reducing ROS accumulation and LDH release, indicating that it alleviated IFN-β-induced oxidative stress and cellular injury in HSkMCs.

These observations are consistent with the findings of Pan et al., who reported that baicalin attenuated oxidative stress, mitochondrial dysfunction, and cell death in an H_2_O_2_-induced C2C12 myoblast injury model (57). Previous studies in macrophages have further shown that baicalin can improve mitochondrial homeostasis, reduce mitochondrial Z-DNA formation, and inhibit the assembly of ZBP1-containing PANoptosomes, thereby alleviating systemic inflammation and tissue injury (24, 58). Our findings extend the potential relevance of this mechanism beyond macrophages and suggest that baicalin may also modulate ZBP1-associated inflammatory cell death signaling in IFN-β-treated skeletal muscle cells. Nevertheless, differences in cellular context should be considered, and the present results do not establish that the mechanisms identified in macrophages are fully conserved in HSkMCs. Taken together, baicalin may protect HSkMCs through two complementary mechanisms. First, its antioxidant and mitochondria-protective effects may reduce endogenous stress signals capable of promoting ZBP1 activation. Second, its direct association with ZBP1 may weaken the recruitment or retention of downstream cell death-related proteins, including RIPK3, caspase-8, and caspase-6. The convergence of these effects may attenuate ZBP1-associated PANoptosis signaling and thereby limit IFN-β-induced skeletal muscle cell injury.

This study has several limitations. First, the clinical validation cohort was relatively small, and treatment status and PBMC composition may have affected candidate gene expression, warranting validation in larger independent cohorts. Second, the protective effects of baicalin were evaluated mainly in short-term *in vitro* experiments, and its long-term efficacy, *in vivo* effects, and safety remain unclear. Third, the current data mainly support IFN-β-induced PANoptosis-like injury and PANoptosis-associated signaling. Additional evidence, including cleavage of caspase-3, caspase-7, and PARP, as well as pathway-specific genetic or pharmacological interventions, is needed to further define the contribution of individual cell death pathways. Moreover, Co-IP analyses were not performed under ZBP1 knockdown conditions, and the association between ZBP1 and RIPK1 was not examined. Finally, the predicted binding of baicalin to an RHIM-overlapping pocket of ZBP1 requires further validation using domain deletion, site-directed mutagenesis of key residues, and biophysical binding assays.

## 5 Conclusion

In conclusion, integrative transcriptomic and machine-learning analyses identified four candidate core PANoptosis-related genes, ZBP1, CD14, TLR3, and TNFSF10, and generated a four-gene PANoptosis-related risk score with strong discriminatory performance for DM. Clinical validation showed that ZBP1 expression was elevated in peripheral blood mononuclear cells and positively associated with serum creatine kinase levels. Functional studies further indicated that ZBP1 contributes to IFN-β-induced PANoptosis-like injury in HSkMCs by associating with RIPK3, caspase-8, and caspase-6 and promoting coordinated cell death-related signaling. Baicalin directly bound ZBP1, reduced its association with downstream proteins, and attenuated cellular injury. Collectively, these findings identify ZBP1-associated PANoptosis signaling as a potential link between immune activation and skeletal muscle damage in DM and provide a rationale for further investigation of PANoptosis-based molecular stratification and ZBP1-targeted interventions.

## Data Availability

The raw data supporting the conclusions of this article will be made available by the authors, without undue reservation.
