# Supplementary material for: ZBP1 contributes to IFN-β-induced PANoptosis-related skeletal muscle cell injury in dermatomyositis
Source: Front Immunol. 2026 Sep 24;17:1955536. doi: 10.3389/fimmu.2026.1955536 (PMC13645519; doi:10.3389/fimmu.2026.1955536)
Supplement: Supplementary file 1 [file Table1.docx]

**Supplementary Table 1**. List of genes related to PANoptosis

| Number | Gene |
| --- | --- |
| 1 | ADAR |
| 2 | MEFV |
| 3 | AIM2 |
| 4 | NLRP1 |
| 5 | NLRC4 |
| 6 | NLRP9 |
| 7 | NLRP3 |
| 8 | ZBP1 |
| 9 | TNFRSF1A |
| 10 | PYCARD |
| 11 | FADD |
| 12 | CASP10 |
| 13 | CASP1 |
| 14 | CASP2 |
| 15 | CASP12 |
| 16 | CASP4 |
| 17 | CASP3 |
| 18 | CASP6 |
| 19 | CASP5 |
| 20 | CASP7 |
| 21 | DFNA5 |
| 22 | CASP8 |
| 23 | MLKL |
| 24 | GSDMD |
| 25 | RIPK3 |
| 26 | RIPK1 |
| 27 | NAIP |
| 28 | TNF |
| 29 | NLRP6 |
| 30 | GSDMA |
| 31 | GSDMC |
| 32 | GSDMB |
| 33 | APAF1 |
| 34 | BAK |
| 35 | BAX |
| 36 | DIABLO |
| 37 | DCN |
| 38 | CASP9 |
| 39 | FAS |
| 40 | TLR3 |
| 41 | FASLG |
| 42 | BAK1 |
| 43 | CHMP2A |
| 44 | CHMP2B |
| 45 | CHMP3 |
| 46 | CHMP4A |
| 47 | CHMP4B |
| 48 | CHMP4C |
| 49 | CHMP6 |
| 50 | CHMP7 |
| 51 | CYCS |
| 52 | ELANE |
| 53 | GSDME |
| 54 | GZMB |
| 55 | HMGB1 |
| 56 | IL18 |
| 57 | IL1A |
| 58 | IL1B |
| 59 | IRF1 |
| 60 | IRF2 |
| 61 | TP63 |
| 62 | AIFM1 |
| 63 | AKT3 |
| 64 | APPL1 |
| 65 | BMF |
| 66 | BNIP3L |
| 67 | BOK |
| 68 | CD14 |
| 69 | CHUK |
| 70 | CRADD |
| 71 | DFFA |
| 72 | DFFB |
| 73 | E2F1 |
| 74 | HMGB2 |
| 75 | IGF1 |
| 76 | LY96 |
| 77 | PPP3R1 |
| 78 | TFDP1 |
| 79 | TICAM1 |
| 80 | TNFSF10 |
| 81 | TP73 |
| 82 | TRAF2 |
| 83 | UACA |
| 84 | UNC5B |
| 85 | YWHAE |
| 86 | YWHAG |
| 87 | MAP3K7 |
| 88 | TNFAIP3 |
| 89 | RNF31 |
| 90 | RBCK1 |
| 91 | PSTPIP2 |
| 92 | TAB3 |
| 93 | PARP1 |
| 94 | TRADD |
| 95 | TAB2 |
| 96 | NR2C2 |
| 97 | AXL |
| 98 | CFLAR |
| 99 | GPX4 |
| 100 | GZMA |
| 101 | IL6 |
| 102 | NLRP7 |
| 103 | NOD1 |
| 104 | PLCG1 |
| 105 | PRKACA |
| 106 | TLR4 |
| 107 | TNFRSF10A |
| 108 | TNFRSF10B |
| 109 | TP53 |
