# Supplementary material for: ZBP1 contributes to IFN-β-induced PANoptosis-related skeletal muscle cell injury in dermatomyositis
Source: Front Immunol. 2026 Sep 24;17:1955536. doi: 10.3389/fimmu.2026.1955536 (PMC13645519; doi:10.3389/fimmu.2026.1955536)
Supplement: Supplementary file 2 [file Table2.docx]

**Supplementary Table 2**. Baseline characteristics of patients with dermatomyositis and healthy controls

| Characteristics | DM (n=25) | HC (n=15) | P value |
| --- | --- | --- | --- |
| Age, years | 54.44 ± 14.20 | 48.53 ± 9.59 | 0.165 |
| Sex, female, n (%) | 17 (68) | 9 (60) | 0.608 |
| Neutrophil count (×10⁹/L) | 4.046 ± 1.805 | 3.816 ± 0.961 | 0.603 |
| Monocyte count (×10⁹/L) | 0.494 ± 0.209 | 0.412 ± 8.68 | 0.104 |
| Glucocorticoid treatment, n (%) | 13 (52) | NA | - |
| Immunosuppressive therapy, n (%) | 7 (28) | NA | - |

Note: Dermatomyositis, DM; Healthy control, HC.
