# Supplementary material for: ZBP1 contributes to IFN-β-induced PANoptosis-related skeletal muscle cell injury in dermatomyositis
Source: Front Immunol. 2026 Sep 24;17:1955536. doi: 10.3389/fimmu.2026.1955536 (PMC13645519; doi:10.3389/fimmu.2026.1955536)
Supplement: Supplementary file 3 [file Table3.docx]

**Supplementary Table 3**. siRNA-ZBP1 primer sequence.

| Gene | Amplicon Size  （bp） | Forward primer  （5'→3'） | Reverse primer  （5'→3'） |
| --- | --- | --- | --- |
| Hu-β-actin | 96 | CCCTGGAGAAGAGCTACGAG | GGAAGGAAGGCTGGAAGAGT |
| Hu-ZBP1 | 195 | TCCATTGCAAACTCCGAAGC | CCGTCTCAGTATGGACTGCT |
| (NC) |  | UUCUCCGAACGUGUCACGUTT | ACGUGACACGUUCGGAGAATT |
| ZBP1-siRNA-253(Si1) |  | AGGAAGACAUCUACAGGUUTT | AACCUGUAGAUGUCUUCCUTT |
| AARS1-siRNA-302(Si2) |  | GGGAAUGAGGACAGCAAAATT | UUUUGCUGUCCUCAUUCCCTT |
| AARS1-siRNA-426(Si3) |  | GCAAAGUCAGCCUCAAUUATT | UAAUUGAGGCUGACUUUGCTT |

Note: β-actin, anti-beta-actin; ZBP1, Z-DNA-binding protein 1.
