# Supplementary material for: ZBP1 contributes to IFN-β-induced PANoptosis-related skeletal muscle cell injury in dermatomyositis
Source: Front Immunol. 2026 Sep 24;17:1955536. doi: 10.3389/fimmu.2026.1955536 (PMC13645519; doi:10.3389/fimmu.2026.1955536)
Supplement: Supplementary file 4 [file Table4.docx]

**Supplementary Table 4**. mRNA sequence used in this study

| Gene | Amplicon Size  （bp） | Forward primer  （5'→3'） | Reverse primer  （5'→3'） |
| --- | --- | --- | --- |
| β-actin | 96 | CCCTGGAGAAGAGCTACGAG | GGAAGGAAGGCTGGAAGAGT |
| ZBP1 | 195 | TCCATTGCAAACTCCGAAGC | CCGTCTCAGTATGGACTGCT |
| CD14 | 100 | TAGACCTCAGCCACAACTCG | CCAGCGAACGACAGATTGAG |
| TNFSF10 | 155 | TGAAGCAGATGCAGGACAAGT | TCTTGGAGTCTTTCTAACGAGC |
| TLR3 | 117 | GCCATGAAGTTGCTGACTGC | TGGCGGCTGGTAATCTTCTG |

Note:β-actin, anti-beta-actin; ZBP1, Z-DNA binding protein 1; CD14, CD14 molecule; TNFSF10, tumor necrosis factor superfamily member 10; TLR3, toll-like receptor 3.
